# Pediatric health-related quality of life and school social capital through network perspectives

**DOI:** 10.1371/journal.pone.0242670

**Published:** 2020-12-02

**Authors:** Tomoya Hirota, Michio Takahashi, Masaki Adachi, Kazuhiko Nakamura

**Affiliations:** 1 Department of Psychiatry and Behavioral Sciences, Weill Institute for Neurosciences, University of California San Francisco, San Francisco, CA, United States of America; 2 Department of Neuropsychiatry, Graduate School of Medicine, Hirosaki University, Hirosaki, Aomori, Japan; 3 Graduate School of Health Sciences, Hirosaki University, Hirosaki, Aomori, Japan; 4 Research Center for Child Mental Development, Graduate School of Medicine, Hirosaki University, Hirosaki, Aomori, Japan; Chiba Daigaku, JAPAN

## Abstract

**Background:**

Despite their importance in population health among children and adolescents, our understanding of how individual items mutually interact within and between pediatric health-related quality of life (HRQOL) and school social capital is limited.

**Methods:**

We employed network analysis in a general population sample of 7759 children aged 9–15 years to explore the network structure of relations among pediatric HRQOL and school social capital items measured using validated scales. Furthermore, network centrality was examined to identify central items that had stronger and more direct connections with other items in the network than others. Network structure and overall strength of connectivity among items were compared between groups (by sex and age).

**Results:**

Our analysis revealed that the item related to school/academic functioning and the item related to shared enjoyment among students had the highest strength centrality in the network of HRQOL and school social capital, respectively, underpinning their critical roles in pediatric HRQOL and school social capital. Additionally, the edge connecting “I trust my friends at school” and “trouble getting along with peers” had the strongest negative edge weight among ones connecting school social capital and pediatric HRQOL constructs. Network comparison test revealed stronger overall network connectivity in middle schoolers compared to elementary schoolers but no differences between male and female students.

**Conclusion:**

The network approach elucidated the complex relationship of mutually influencing items within and between pediatric HRQOL and school social capital. Addressing central items may promote children’s perceived health and school social capital.

## Introduction

Pediatric health-related quality of life (HRQOL) is a multi-dimensional construct, encompassing children's physical, emotional, and social functioning, perceived health, and well-being [1, 2]. Self-assessed health status measured via HRQOL is reported to predict mortality and morbidity [3]. It is considered a measure of population health and thus has been used in studies related to health promotion (e.g., Healthy People: https://www.cdc.gov/hrqol/concept.htm#). Studies pertaining to pediatric HRQOL have largely focused on clinical populations [4, 5]. However, a study proposed the importance of analyzing HRQOL in a general population of children and adolescents, as measuring generic pediatric HRQOL in addition to disease-specific HRQOL can help better understand pediatric population health [6].

Social capital is a concept reflecting community cohesion, trust, and reciprocity [7] and an important determinant of health [8]. In children and adolescents, the measurement of social capital entails multiple contexts, such as family functioning, peer relationship, and school climate [9, 10]. Among them, school contexts are critical for early adolescents as individuals at this developmental stage form relationships with peers and adults/teachers in school [11].

Several existing studies have indicated the association between school social capital and pediatric HRQOL. For example, a study conducted among 2^nd^, 3^rd^, and 5^th^ grade students in the U.S. revealed the association of school connectedness with their HRQOL scores and underscored school connectedness (a student’s belief that people in school care about their learning and them as individuals) as a potential modifiable factor for enhancing students’ perceived health [12]. In previous studies conducted among a general population sample of 7^th^– 12^th^ students in the U.S., students who felt connected to school were more likely to engage in healthy behaviors and succeeded academically [13]. The mechanism accounting for the relationship between school connectedness and students’ HRQOL remains unknown. However, in one exploratory study, authors demonstrated that school connectedness partially mediated the association between emotional distress and school engagement which pertained to educational attainment and school performance [14]. Additionally, other studies also reported the association of other indices of school social capital, such as sense of belonging, support from peers and teachers, and safety, with HRQOL [15–17]. Studies have suggested that these indices are malleable and thus can be modifiable factors [18, 19]. Thus, the development of school-based interventions targeted for students with weak school social capital could potentially promote their HRQOL.

In health science research, researchers have traditionally conceptualized HRQOL at the construct or domain level. This conceptualization is based on the model that HRQOL is an unobservable/a latent entity that is the common effect of variables/items. However, this model does not reflect an intricate system of mutually interacting item-item associations that form HRQOL. Given its role in population health and other health sciences, as described above, a plausible approach to the constructs of HRQOL is needed. To overcome the methodological challenge, network analysis was previously employed in a general population sample of Dutch adults, which supported its utility for HRQOL research [20]. Notwithstanding, no studies using this approach have investigated the network structure of HRQOL in pediatric populations. Furthermore, despite the complex relations between contextual effects of school social capital on HRQOL, no studies have examined unique associations between these constructs using the network approach.

Therefore, in this study, we first proposed to examine the network architecture of pediatric HRQOL and school social capital in 4th– 9^th^ grade Japanese students aged 9–15 years. Second, we aimed to identify central items that had strong connections with other items and were also highly influential within that network. Furthermore, we aimed to determine important item-item relations between these two constructs. Additionally, as previous studies reported the association of age and sex with school social capital and HRQOL [21, 22], we also aimed to compare the network structures between 4th– 6^th^ graders (elementary school students) and 7^th^– 9^th^ graders (middle school students) and between boys and girls.

## Methods

### Ethics approval

The protocol of the current study was approved by the Committee on Medical Ethics of Hirosaki University (IRB# 2015–055). The current study was conducted in accordance with the ethical standards laid down in the 1964 Declaration Helsinki and its later amendments. The written consent to the study participation was obtained from caregivers of children and adolescents who participated in the school survey.

### Study setting and participants

We conducted a community-based survey in September 2018, targeting children between 4^th^ and 9^th^ grades in national and public elementary (4^th^– 6^th^ graders) and junior high/middle schools (7^th^– 9^th^ graders) in Hirosaki city, Japan. First, we mailed letters to caregivers of each student and provided information about the survey. Students whose caregivers did not consent to the survey were excluded from this study. We distributed 8184 sets of questionnaires to the corresponding schools. Students were briefed about the purpose of the survey and completed the questionnaire in the classroom. A total of 425 students were excluded from data analysis due to missing data, yielding 7759 students in total (3909 males; 50.4% and 3850 females; 49.6%) (**Table 1**). Comparison of participating patients with excluded patients did not reveal significant differences in characteristics described in Table 1.

**Table 1:** Characteristics of 7759 students included in network analysis.

	male	female	*p*	4–6th grade	7–9th grade	*p*
N (%)	3909 (50.4)	3850 (49.6)		3760 (48.5)	3999 (51.5)	
PedsQL raw scores (mean (SD))	13.30 (13.04)	12.77 (12.26)	0.065	15.91 (12.77)	10.33 (11.94)	< 0.001
PedsQL scale scores (mean (SD))	85.55 (14.17)	86.12 (13.32)	0.065	82.71 (13.88)	88.77 (12.98)	< 0.001
SCQ-AS school social capital subscale score (mean (SD))	20.62 (3.08)	20.67 (2.88)	0.410	20.59 (2.92)	20.69 (3.04)	0.149

Abbreviations: PedsQL, Pediatric Quality of Life Inventory 4.0 generic scale; SCQ-AS, Social Capital Questionnaire for Adolescent Students.

### Measures

The participants’ HRQOL was measured using Pediatric Quality of Life Inventory TM (PedsQL TM) Generic Core Scale [23]. The PedsQL is a 23‐item instrument and consists of four domains: physical (eight items), emotional (five items), social (five items), and school (five items) functioning. The earlier study demonstrated good reliability and validity of this instrument in the U.S. sample [24]. The Japanese-version of the PedsQL was developed and standardized in a general population sample of Japanese children and adolescents [25]. In this study, participants were asked how much of a problem each item had been for them the past one month, ranging from “never” to “almost always a problem.” **Table 2** lists the individual items of the PedsQL.

**Table 2:** Descriptions of the individual items used for network analysis.

Item/node number	Domain	Node name	Item/node description
***Pediatric HRQOL***			
1	Physical Functioning (PF)	PF1	Hard to walk more than one block
2		PF2	Hard to run
3		PF3	Hard to do sports or exercises
4		PF4	Hard to lift something heavy
5		PF5	Hard to take bath or shower
6		PF6	Hard to do chores around the house
7		PF7	Hurt or ache
8		PF8	Low energy
9	Emotional Functioning (EF)	EF1	Feel afraid or scared
10		EF2	Feel sad or blue
11		EF3	Feel angry
12		EF4	Trouble sleeping
13		EF5	Worry about what will happen
14	Social Functioning (SoF)	SoF1	Trouble getting along with peers
15		SoF2	Other kids not wanting to be friend
16		SoF3	Teased
17		SoF4	Doing things other peers do
18		SoF5	Hard to keep up when play with others
19	School Functioning (ScF)	ScF1	Hard to concentrate
20		ScF2	Forget things
21		ScF3	Trouble keeping up with schoolwork
22		ScF4	Miss school—not well
23		ScF5	Miss school—doctor appointment
***School social capital***			
24	School social capital (SSC)	SSC1	Students stay together
25		SSC2	Feel belonging at school
26		SSC3	Feel safe at school
27		SSC4	Parents get along with teachers
28		SSC5	Students share enjoyment
29		SSC6	Trust friends at school
30		SSC7	Can ask friends at school for help
31		SSC8	Teachers are supportive

School social capital was measured using eight items of the Social Capital Questionnaire for Adolescent Students (SCQ-AS) [26]. The SCQ-AS is a self-administered 12-item questionnaire and was standardized in a general population of Japanese students [27]. Factor analysis of this scale produced three factors [27]; the eight items selected for the measurement of school social capital in this study belonged to the factor “school social cohesion and network” (**Table 2**). The internal consistency of this factor in our participants was Cronbach’s α = 0.81.

### Analytic plans

#### Network estimation

In this study, we estimated a Gaussian graphical model using the matrix of polychoric correlations between 31 items of the PedsQL and the SCQ-AS (23 pediatric HRQOL items and 8 school social capital items).The Gaussian graphical model is a regularized partial correlation network to model the association between different constructs or components. In the network model, individual variables are defined as nodes and relationships between nodes are defined as edges. In this model, lack of edge between two nodes means conditional independence relationships among the nodes. Edge thickness/weights can be understood as an estimation of partial correlations coefficients, representing the correlation between two nodes when controlling for all other nodes in the network [28]. The network was estimated and visualized using the R-package “qgraph” [29]. To reduce the likelihood of type-I errors, “qgraph” employs the EBICglasso procedure, leading to a parsimonious network with explanatory power. Additionally, we used the walktrap community detection algorithm [30] to explore how highly associated clusters of symptoms were nested within the broader network (see **S1 Data** for details about this procedure). Missing data were handled using listwise deletion.

#### Centrality

We calculated three commonly used indices of node centrality to quantify the importance of each item in the network [31]. Degree strength is determined by summing edge weights in a weighted network. Closeness is another centrality index that can be calculated by taking the inverse of the sum of distances of the node of interest from all other nodes in the whole network. Lastly, betweenness refers to the number of times a node lies on the shortest path between two other nodes. These indices are presented as standardized z-scores, with higher values reflecting greater overall importance of a symptom to the network. As strength centrality is generally more stable than betweenness and closeness [32], we considered the item with the highest strength centrality the central item in the network.

#### Network accuracy

Edge weight accuracy and centrality stability were measured using bootstrapped difference tests and case-dropping subsampling procedures (see Epskamp et al [32] for details of this procedure). They refer to the degrees of confidence when interpreting the rank ordering of the edge weights and central values.

#### Network comparison

Developmental and sex differences were explored by splitting the sample by sex and age and estimating separate networks for each group. Networks from different groups were compared in overall structural invariance (whether the structure is identical across groups or not) and global connectivity invariance (whether symptoms are connected to the same degree across groups or not) using the “NetworkComparisonTest” [33]. We compared networks between males and females and between 4th– 6^th^ graders and 7^th^– 9^th^ graders using non-parametric permutation tests (1000 random permutations were used in this study).

## Results

**Table 1** represents characteristics of 7759 students who completed the questionnaire with no missing data on HRQOL and social capital items.

### Network estimation

**Fig 1:**
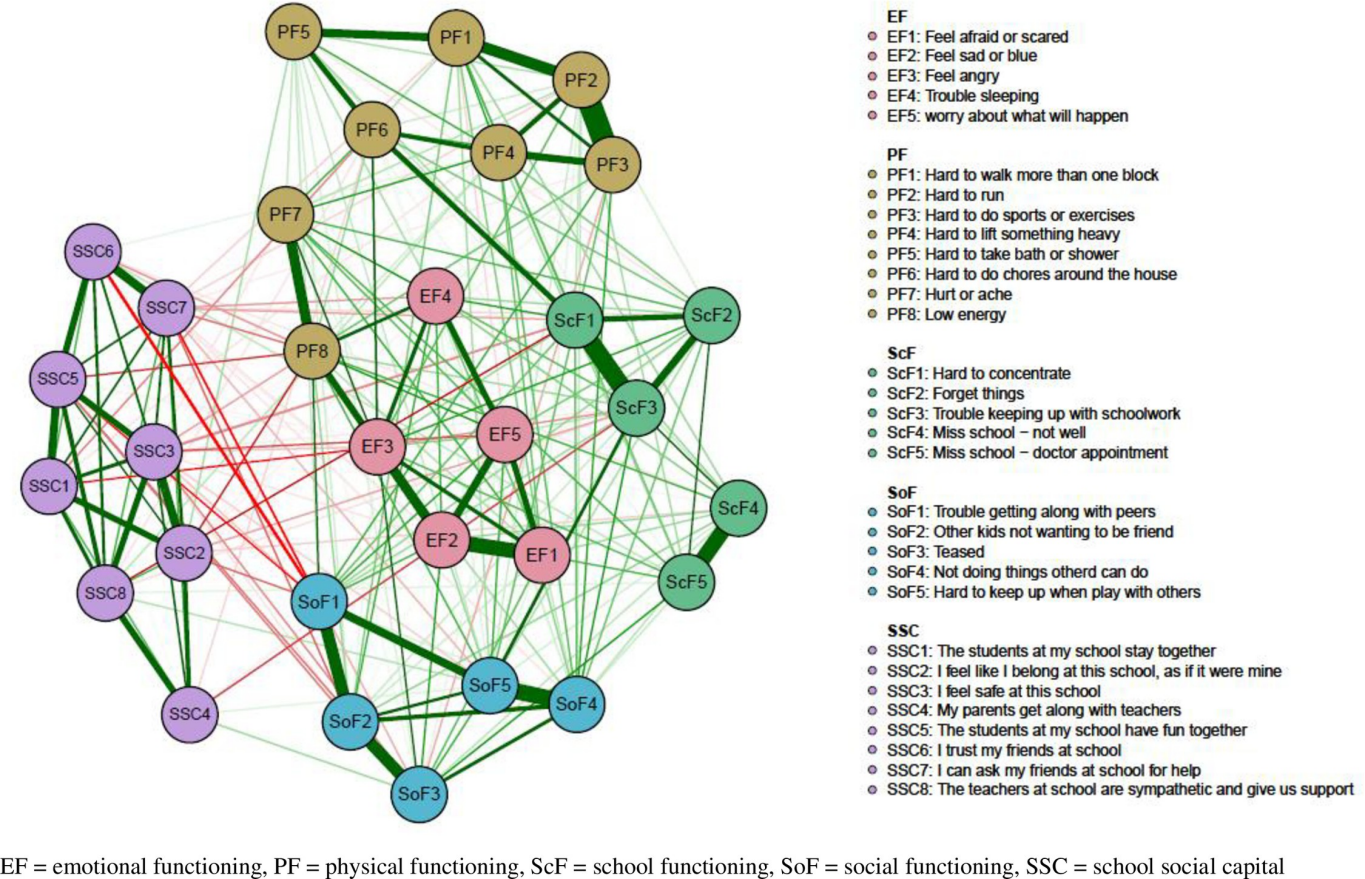
School social capital and health-related quality of life (HRQOL) network.

**Fig 1** depicts the network structure of relations among school social capital and HRQOL items. HRQOL items formed four major HRQOL clusters, which seemed consistent with the four domains defined in the PedsQL (physical, emotional, school, and social functioning). However, two items, “hurt or ache (PF7)” and “low energy (PF8)” that were originally constructed in the physical functioning domain did not cluster with other physical functioning items in the network. Instead, these two items were located adjacent to items belonging to the emotional functioning domain, and “low energy” item had a relatively strong association with “feel sad or blue (EF2)” item. The walktrap community detection algorithm applied to the network above supported the four-community interpretation, where “hurt or ache” and “low energy” items were grouped in the emotional functioning community (**S2 Data**). HRQOL items were overall strongly associated with other items in the same cluster (all edge weights of this network are listed in **S3 Data)**.

Focusing on the item-item relations between school social capital and HRQOL, 80% (52/65) of the edges connecting between social capital and HRQOL had negative weights, suggesting potential protective roles of school social capital in adolescents’ HRQOL. Among them, the edge connecting “I trust my friends at school (SSC6)” and “trouble getting along with peers (SoF1)” had the strongest negative edge weight (edge weight = -0.11).

### Network centrality

**Fig 2:**
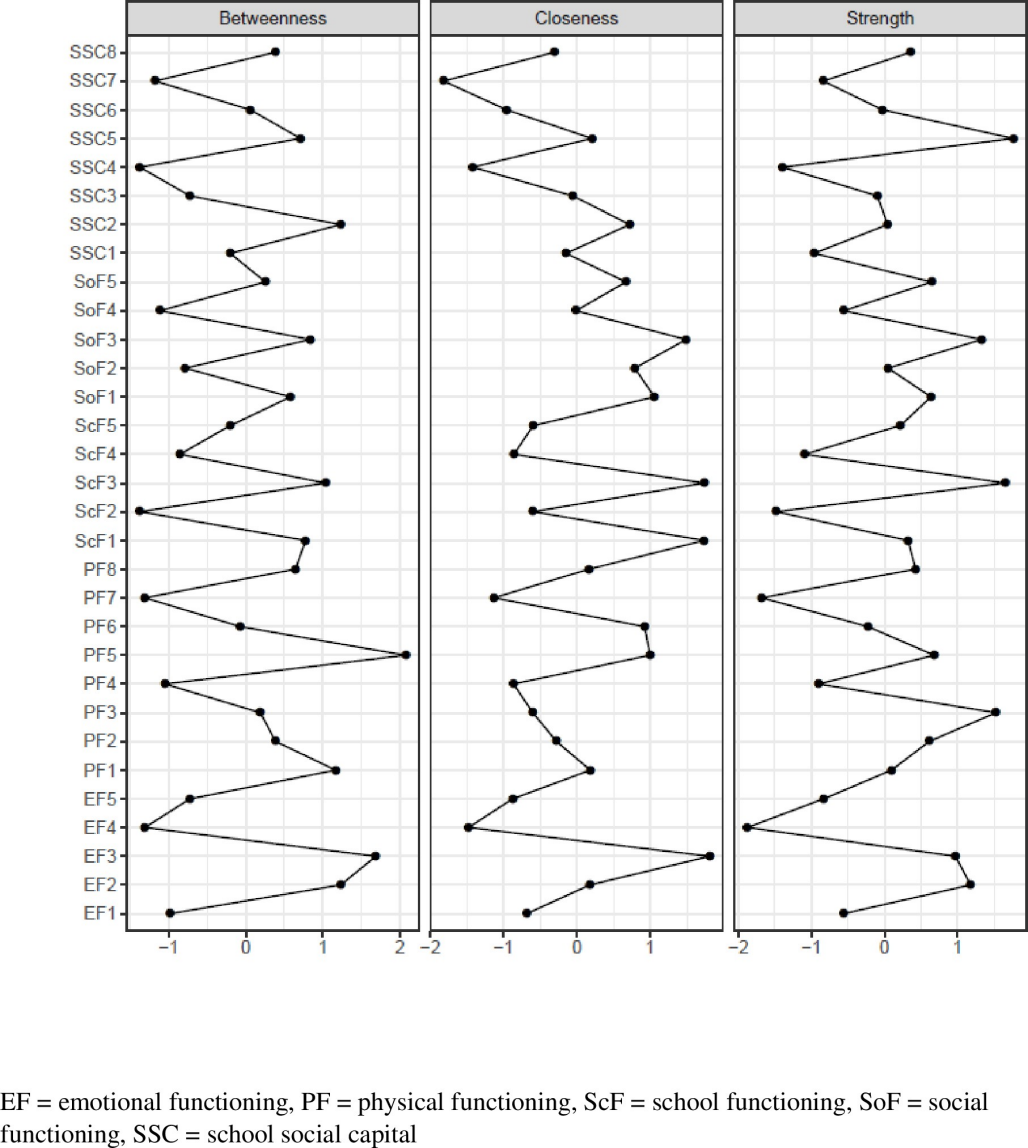
Network centrality among school social capital and HRQOL.

**Fig 2** represents plots depicting centrality indices of 33 items to the whole network of HRQOL and school social capital. Focusing on HRQOL items, the item “trouble keeping up with school (ScF3)” was the highest in strength, suggesting the critical role of school/academic functioning in adolescents’ overall HRQOL. In addition, this item had high closeness. As seen in Fig 1, this item was influential in connecting other symptoms that were otherwise unrelated in the school functioning cluster. Other important items were “teased (SoF3),” “feel sad or blue (EF2),” and “feel angry (EF3),” that were high in two or more central indices. Regarding school social capital, the item “the students have fun together (SSC5)” (shared enjoyment) was highest in strength and the item “I feel like I belong at this school (SSC2)” (sense of belonging) was high in betweenness.

### Network accuracy

Examination of the network accuracy revealed that the rank ordering of edge weights was considered accurate as bootstrap CIs of the strong edges did not overlap zero (**S4 Data**). Strength and closeness were considered stable as the correlation stability coefficients for those centrality indices in this group were above 0.5 (strength: 0.75, closeness: 0.52, betweenness: 0.36, **S5 Data**) [34].

### Network comparison

Network comparison between male and female students revealed no statistically significant differences for the overall network structure (*p*  =  0.18) and global connectivity (p = 0.89) for the two networks, suggesting that the network structures did not significantly differ by sex.

However, network comparison between elementary (4^th^– 6^th^ graders) and middle school students (7^th^– 9^th^ graders) showed statistically significant differences for both values (overall network structure: *p* = 0.001, global connectivity: *p* = 0.03). Connectivity values were larger in middle school students (13.83) than that of elementary school students (13.38). Post-hoc comparisons of each edge weight between the two networks revealed 16 specific edge weights that reached statistical significance (**S6 Data**).

## Discussion

In this study, we employed network analysis to examine item-item associations of pediatric HRQOL and school social capital among Japanese adolescents in a general population. This is the first study using the network approach on these constructs in pediatric populations, and thus, our findings are exploratory. Our analysis showed that the school functioning item and the item related to shared enjoyment among students had the highest strength centrality in the network of HRQOL and school social capital, respectively. Furthermore, the edge connecting “I trust my friends at school” and “trouble getting along with peers” had the strongest negative edge weight among ones connecting school social capital and pediatric HRQOL. The network comparison test revealed stronger overall network connectivity in middle schoolers compared to that in elementary schoolers, but no differences in overall network connectivity between male and female students.

Within the HRQOL network, it was notable that pain and fatigue items, originally constructed in the physical functioning domain in the scale development process, belonged to the emotional functioning community/cluster with other emotion items (e.g., anger, sadness, fear) in the network (**Fig 1** and **S2 Data**). The association between pain and emotion was examined by previous non-network studies [5, 35]; one study reported that the correlation of pain with psychological/emotional wellbeing in a general population sample of children was stronger with that of physical wellbeing, corroborating our findings [5]. In **Fig 1**, the pain item was weakly connected with emotion items, while the fatigue item had relatively strong associations with emotion items that were related to other domain items in the HRQOL network, suggesting potential mediating roles of fatigue in the relationship between pain and the overall HRQOL. The mediating role of fatigue in the relations between pain and HRQOL was proposed in previous studies with certain diseases [36, 37].

In the HRQOL network, the item “trouble keeping up with schoolwork (ScF3)” was considered central to the network due to its high centrality indices, highlighting the importance of academic and learning functioning in pediatric HRQOL. This item was strongly associated with the adjacent school functioning items (“hard to concentrate (ScF1)” and “forget things (ScF2)”) in the network, implying a contribution of attention difficulty to learning/academic challenges, poor school functioning, and overall low HRQOL. In fact, children with ADHD had poorer HRQOL in comparison to the normative US and Australian pediatric samples [38]. Thus, our finding indicates that the assessment for students’ learning and attention abilities is essential to promote better wellbeing.

Two emotion items, “feel angry (EF3)” and “feel sad or blue (EF2)” also had high centrality indices, indicating the magnificent role of emotional problems in the pediatric HRQOL. This finding is consistent with studies reporting lower HRQOL in clinical samples of adolescents (e.g., epilepsy [39]) who exhibited emotional difficulty, including depression. Adolescence is the pivotal period for the development of emotion regulation in the face of increasing daily life stressors and exposure to negative emotions [40]. Poor emotion regulation is considered a transdiagnostic risk factor for a wide range of psychopathological outcomes [41, 42], and thus, future research should examine the effect of interventions, such as emotion regulation training, on adolescents wellbeing.

Our results suggested the importance of shared enjoyment among students at school (the item “the students have fun together (SSC5)”) based on its highest strength among school social capital items. As shows in **Fig 1**, this item was strongly associated with other school social capital items, such as cohesiveness among students, safety, trust, and supportive school environment. Therefore, strengthening shared enjoyment among students can have a huge impact on other school social capital items and indirectly promote students’ HRQOL (for example, through the strong edge between “I trust my friends at school (SSC6)” and “trouble getting along with peers (ScF1)”). To support this, one study reported the effectiveness of a positive psychology program that promoted students’ positive engagement and experiences on their HRQOL [43].

Associations between certain school social capital items (e.g., “I trust my friends at school (SSC6),” “the students stay together (SSC1),” and “I feel like I belong at this school (SSC2),”) and HRQOL items are notable. Although the magnitude of these associations was modest, these associations corroborate findings in previous non-network studies. For example, studies have reported that positive perceptions of school connectedness (sense of belonging) and positive relationships with peers and teachers (connectedness, support) were associated with increased psychosocial well-being and decreased adolescent mental health problems [44, 45]. Accordingly, our study findings underpin the gravity of cultivating better school social capital for students’ health promotion.

Network comparison test revealed overall stronger connectivity among HRQOL problems, reflecting lower total HRQOL scores, in middle school students than in elementary school students, implying developmental contributions to the network. The decrease in the total HRQOL scores with age was previously reported. For example, in a study that examined longitudinal changes of pediatric HRQOL from 8 to 18 years of age, the HRQOL scores decreased continually [18]. The significant difference in the network between the two groups can be attributable to the students’ adjustment difficulty in transitioning from elementary to middle school [46]. Among 46 edge weights that reached statistical significance in edge weight between these two groups, we only identified one edge that connected school social capital and HRQOL. Moreover, this edge weight was significantly larger in elementary school students than that of in middle school students, suggesting stronger relationships between positive engagement and HRQOL among elementary school students. A longitudinal study that followed students during their transition from primary school to middle school revealed a decrease in school connectedness and peer support over time [46], supporting our finding in this study.

Some limitations of our study are as follows. First, the cross-sectional design prohibits us from inferring the direction of causality between school social capital and pediatric HRQOL. We assumed that school social capital could have an impact on pediatric HRQOL; however, it is also probable that adolescents with lower HRQOL are more likely to have lower degree of school social capital. Second, we did not obtain data related to socioeconomic status (SES), and thus, we were unable to examine the degree of influence of participants’ SES (household income) on their perceived health status. Third, we did not include structural school social capital, referring to networks and organizations (e.g., students’ participation in extracurricular activities), which are reported to be associated with their well-being [47].

Despite the limitations above, this study has several strengths. This is the first study that examined the item-item associations of pediatric HRQOL and school social capital using network analysis, a novel statistical approach. Additionally, the large sample size in a general population affords statistical powers to examine associations among individual items. Furthermore, the use of students’ self-report is an advantage over the use of parental reports on these constructs as subjective reports on their perceived health and school climates are less likely to lead to misinterpretations.

## Supporting information

S1 Data Walktrap community detection algorithm. (DOCX)

S2 Data HRQOL and school social capital network (walktrap communities). (TIF)

S3 Data Edge weights. (DOCX)

S4 Data Network accuracy. (TIF)

S5 Data Centrality stability. (TIF)

S6 Data Network comparison between elementary school students and middle school students. (TIF)

S7 Data Edge weights with statistical significance by the network comparison test (4^th^–6^th^ graders vs 7^th^–9^th^ graders). (DOCX)
