# Supplementary material for: Pediatric health-related quality of life and school social capital through network perspectives
Source: PLoS One. 2020 Dec 2;15(12):e0242670. doi: 10.1371/journal.pone.0242670 (PMC7710098; doi:10.1371/journal.pone.0242670)
Supplement: S1 Data — (DOCX) [file pone.0242670.s001.docx]

**S1 Data. walktrap community detection algorithm**

The walktrap algorithm is one of the community detection algorithms developed to uncover the dynamic properties of complex networks and to find the number of dense clusters in the network. The simple description of the waltrap algorithm is as follows: 1) starting at a random symptom node, a connection with another node is randomly chosen; 2) this step is repeated multiple times; and 3) random walks get “trapped” in densely connected parts of the network (please see (1) for details).

1. Pons P, Latapy M. Computing Communities in Large Networks Using Random Walks. In: Yolum pInar, Güngör T, Gürgen F, Özturan C, editors. Computer and Information Sciences - ISCIS 2005. Springer Berlin Heidelberg; 2005. p. 284–93. (Lecture Notes in Computer Science).
