# Supplementary figures and images for: Pediatric health-related quality of life and school social capital through network perspectives
Source: PLoS One. 2020 Dec 2;15(12):e0242670. doi: 10.1371/journal.pone.0242670 (PMC7710098; doi:10.1371/journal.pone.0242670)

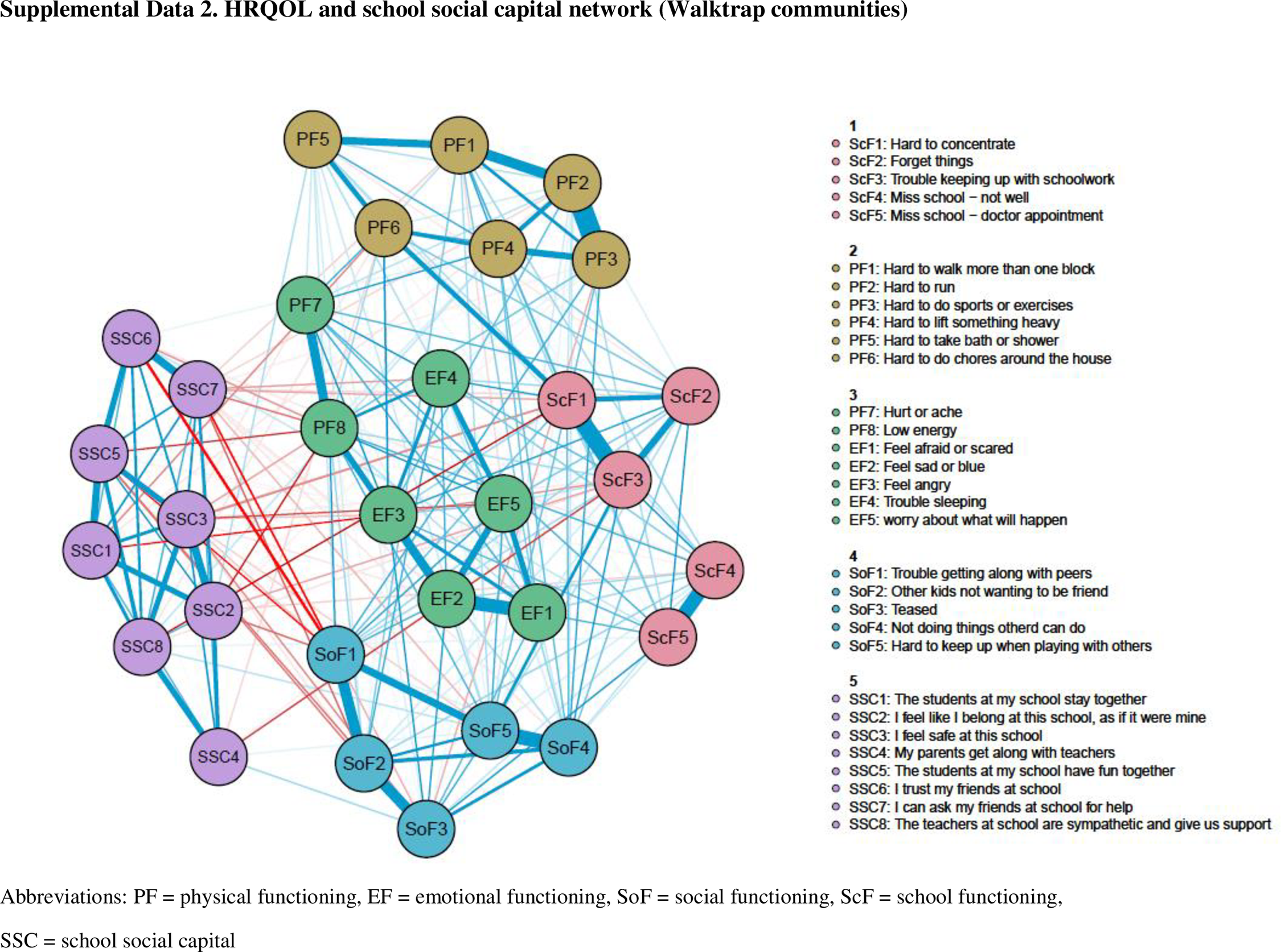

Supplement: S2 Data — (TIF) [file pone.0242670.s002.tif]

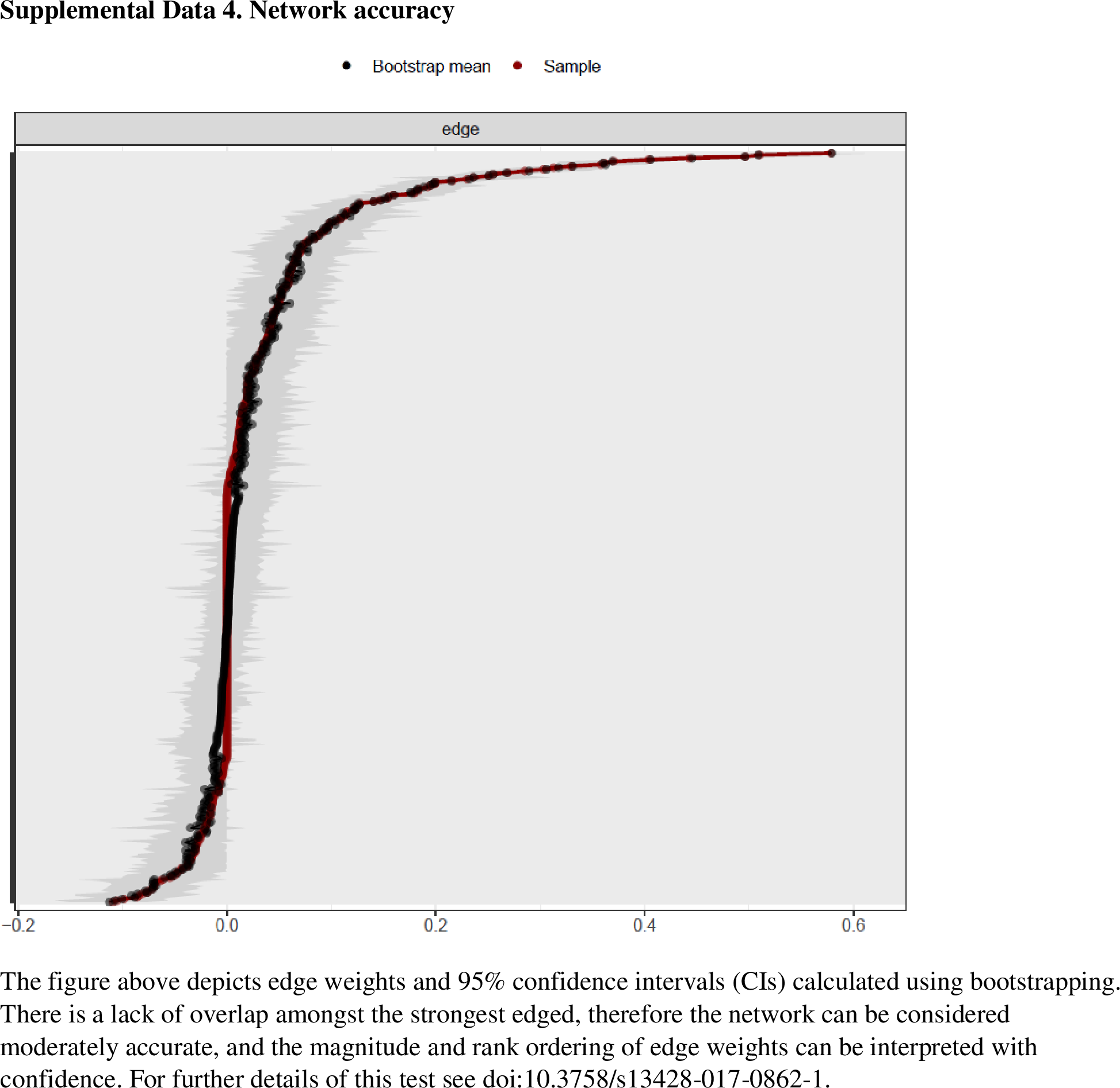

Supplement: S4 Data — (TIF) [file pone.0242670.s004.tif]

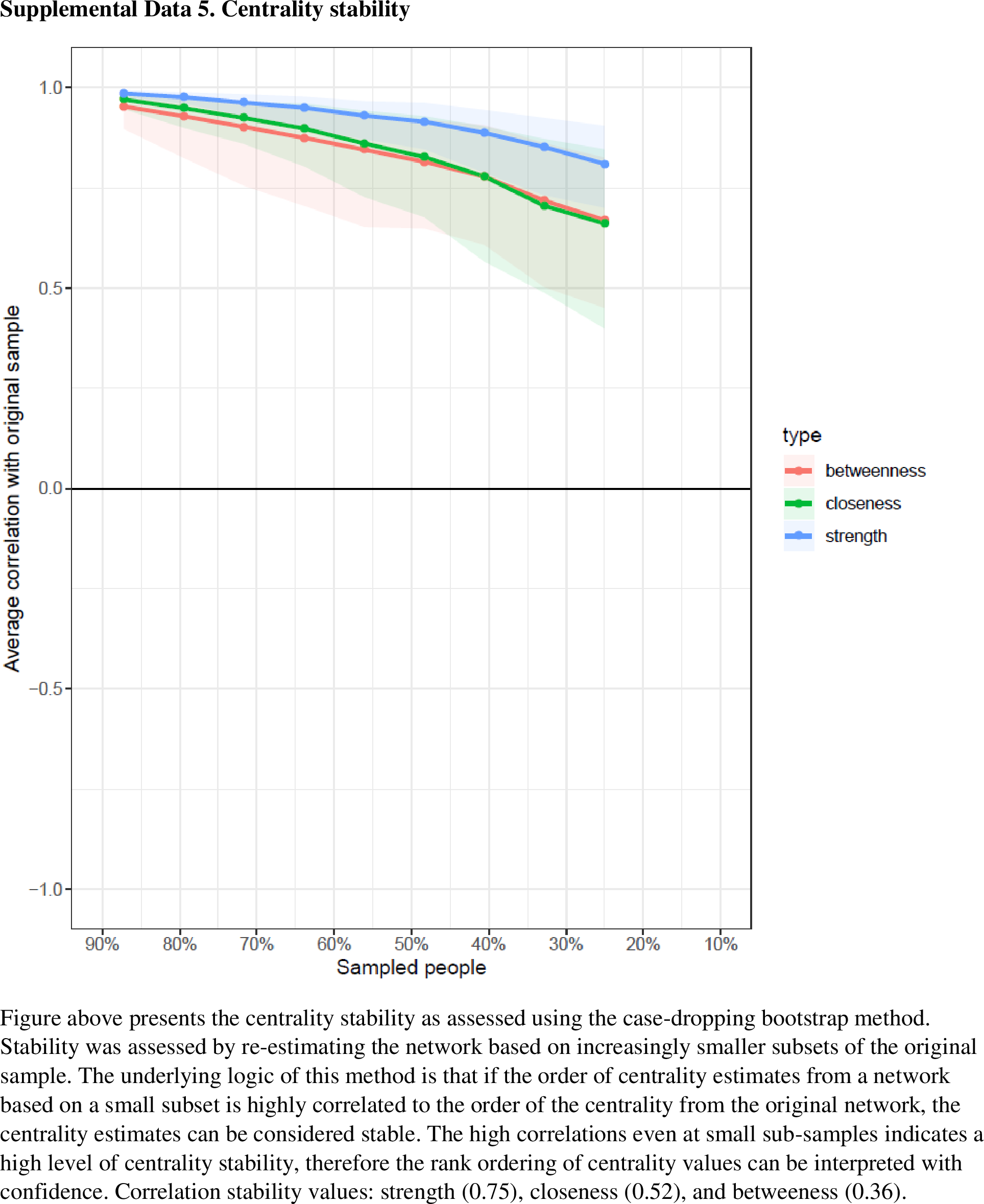

Supplement: S5 Data — (TIF) [file pone.0242670.s005.tif]

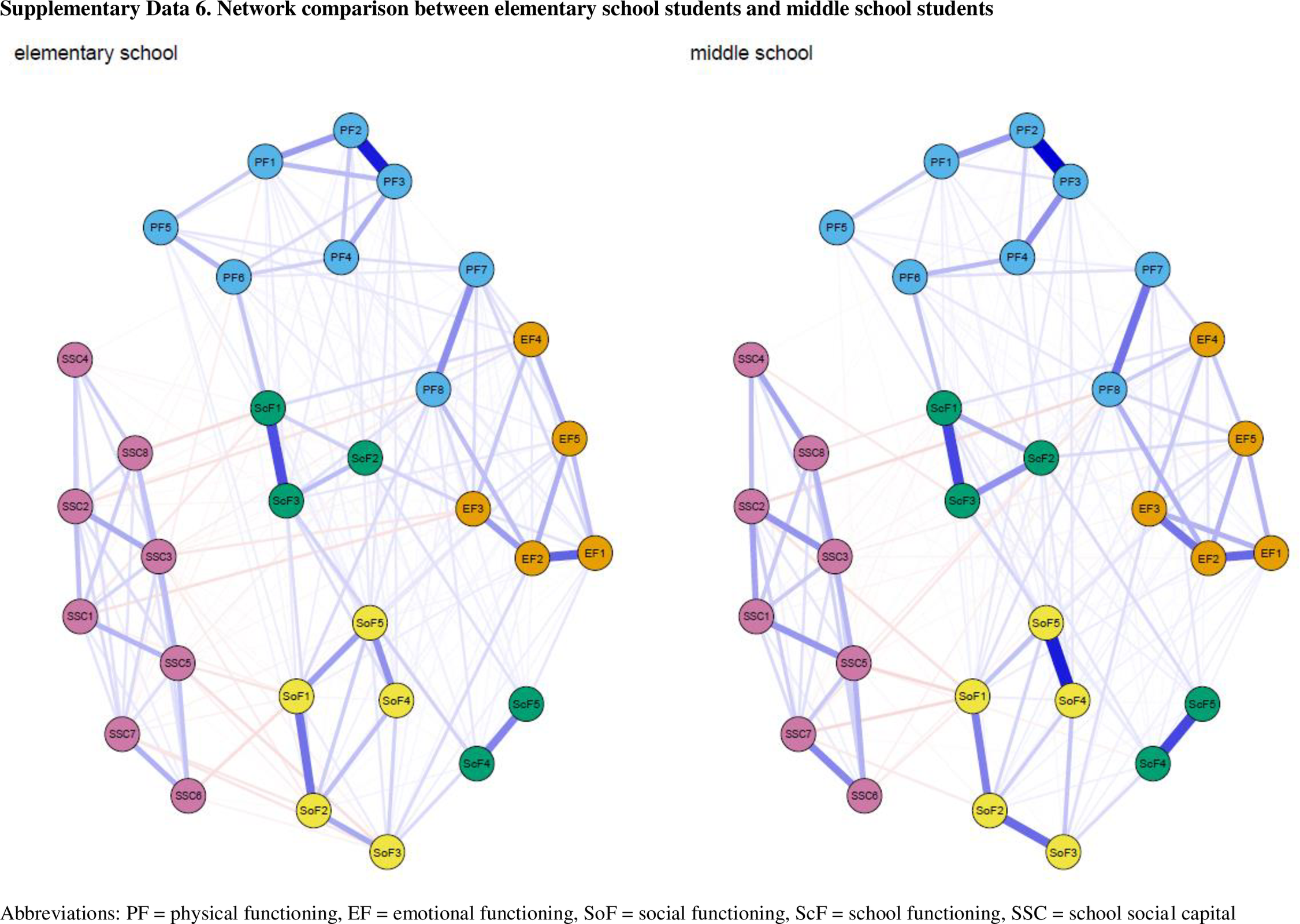

Supplement: S6 Data — (TIF) [file pone.0242670.s006.tif]
