# Supplementary material for: Pediatric health-related quality of life and school social capital through network perspectives
Source: PLoS One. 2020 Dec 2;15(12):e0242670. doi: 10.1371/journal.pone.0242670 (PMC7710098; doi:10.1371/journal.pone.0242670)
Supplement: S3 Data — (DOCX) [file pone.0242670.s003.docx]

**S3 Data. Edge weights**

| From | To | Weight |  | From | To | Weight |  | From | To | Weight |
| --- | --- | --- | --- | --- | --- | --- | --- | --- | --- | --- |
| PF2 | PF3 | 0.58 |  | PF6 | ScF1 | 0.15 |  | EF3 | ScF2 | 0.08 |
| ScF4 | ScF5 | 0.51 |  | ScF1 | ScF2 | 0.15 |  | SSC1 | SSC3 | 0.08 |
| ScF1 | ScF3 | 0.5 |  | SSC1 | SSC2 | 0.14 |  | SSC3 | SSC6 | 0.08 |
| SoF4 | SoF5 | 0.44 |  | EF1 | EF3 | 0.13 |  | PF1 | PF4 | 0.07 |
| EF1 | EF2 | 0.4 |  | SoF2 | SoF4 | 0.13 |  | PF2 | PF4 | 0.07 |
| PF1 | PF2 | 0.37 |  | PF4 | PF5 | 0.12 |  | PF2 | PF5 | 0.07 |
| SoF1 | SoF2 | 0.36 |  | PF4 | PF6 | 0.12 |  | PF5 | EF4 | 0.07 |
| SoF2 | SoF3 | 0.36 |  | EF3 | EF4 | 0.12 |  | PF8 | SoF1 | 0.07 |
| PF7 | PF8 | 0.33 |  | SoF5 | ScF3 | 0.12 |  | EF5 | SoF1 | 0.07 |
| SSC6 | SSC7 | 0.31 |  | ScF3 | ScF4 | 0.11 |  | EF2 | SoF3 | 0.07 |
| SSC1 | SSC5 | 0.3 |  | SoF3 | ScF5 | 0.11 |  | PF3 | SoF4 | 0.07 |
| FP5 | PF6 | 0.29 |  | PF5 | SSC2 | 0.11 |  | EF5 | SoF5 | 0.07 |
| EF2 | EF3 | 0.27 |  | SSC2 | SSC4 | 0.11 |  | SoF2 | SoF5 | 0.07 |
| SSC2 | SSC3 | 0.26 |  | SSC6 | SSC8 | 0.11 |  | EF4 | ScF1 | 0.07 |
| SSC4 | SSC8 | 0.25 |  | PF6 | EF3 | 0.1 |  | SSC1 | SSC7 | 0.07 |
| FPF1 | PF5 | 0.24 |  | PF8 | EF4 | 0.1 |  | PF4 | PF7 | 0.06 |
| PF3 | PF4 | 0.23 |  | SSC3 | SSC4 | 0.1 |  | PF6 | PF7 | 0.06 |
| SoF1 | SoF5 | 0.22 |  | SSC2 | SSC7 | 0.1 |  | PF1 | EF1 | 0.06 |
| EF2 | EF5 | 0.2 |  | SSC1 | SSC8 | 0.1 |  | PF4 | EF1 | 0.06 |
| ScF2 | ScF3 | 0.2 |  | PF1 | PF3 | 0.09 |  | PF2 | EF3 | 0.06 |
| SSC5 | SSC6 | 0.2 |  | EF3 | SoF3 | 0.09 |  | PF7 | EF3 | 0.06 |
| PF8 | EF2 | 0.19 |  | PF1 | ScF5 | 0.09 |  | PF8 | EF3 | 0.06 |
| EF1 | EF5 | 0.18 |  | ScF2 | ScF5 | 0.09 |  | PF7 | EF4 | 0.06 |
| EF4 | EF5 | 0.18 |  | SSC2 | SSC5 | 0.09 |  | PF3 | SoF1 | 0.06 |
| SSC3 | SSC5 | 0.18 |  | SSC4 | SSC7 | 0.09 |  | PF3 | SoF5 | 0.06 |
| SSC3 | SSC8 | 0.18 |  | EF3 | ScF2 | 0.08 |  | PF8 | ScF4 | 0.06 |
| SSC5 | SSC8 | 0.16 |  | SSC1 | SSC3 | 0.08 |  | SoF3 | SSC2 | 0.06 |
| SoF3 | SoF4 | 0.15 |  | SSC4 | SSC7 | 0.09 |  | SoF3 | SSC4 | 0.06 |

**S3 Data. Edge weights (continued)**

| From | To | Weight |  | From | To | Weight |  | From | To | Weight |
| --- | --- | --- | --- | --- | --- | --- | --- | --- | --- | --- |
| SSC3 | SSC7 | 0.06 |  | SoF4 | ScF1 | 0.04 |  | PF7 | EF5 | 0.02 |
| SSC5 | SSC7 | 0.06 |  | SoF4 | ScF2 | 0.04 |  | PF1 | SoF3 | 0.02 |
| SoF5 | SSC8 | 0.06 |  | PF8 | ScF3 | 0.04 |  | PF1 | SoF5 | 0.02 |
| PF3 | PF6 | 0.05 |  | SoF4 | ScF3 | 0.04 |  | EF5 | ScF2 | 0.02 |
| PF3 | PF8 | 0.05 |  | EF2 | ScF4 | 0.04 |  | SoF5 | ScF2 | 0.02 |
| PF7 | EF1 | 0.05 |  | PF7 | ScF5 | 0.04 |  | PF1 | ScF3 | 0.02 |
| PF8 | EF5 | 0.05 |  | EF3 | ScF5 | 0.04 |  | SoF2 | ScF3 | 0.02 |
| EF4 | SoF2 | 0.05 |  | SoF5 | SSC4 | 0.04 |  | EF1 | ScF4 | 0.02 |
| PF8 | SoF5 | 0.05 |  | SoF4 | SSC6 | 0.04 |  | EF4 | ScF4 | 0.02 |
| PF1 | ScF1 | 0.05 |  | PF6 | SSC7 | 0.04 |  | PF2 | ScF5 | 0.02 |
| PF6 | ScF2 | 0.05 |  | SSC7 | SSC8 | 0.04 |  | PF | SSC1 | 0.02 |
| PF5 | ScF4 | 0.05 |  | PF5 | PF7 | 0.03 |  | EF2 | SSC1 | 0.02 |
| EF1 | ScF5 | 0.05 |  | EF3 | SoF4 | 0.03 |  | PF1 | SSC3 | 0.02 |
| PF6 | SSC5 | 0.05 |  | PF4 | ScF2 | 0.03 |  | PF4 | SSC3 | 0.02 |
| ScF5 | SSC6 | 0.05 |  | PF7 | ScF2 | 0.03 |  | EF2 | SSC4 | 0.02 |
| SSC1 | SSC6 | 0.05 |  | SoF1 | ScF2 | 0.03 |  | EF5 | SSC4 | 0.02 |
| SSC2 | SSC6 | 0.05 |  | EF4 | ScF3 | 0.03 |  | ScF2 | SSC5 | 0.02 |
| PF4 | SSC8 | 0.05 |  | EF5 | ScF3 | 0.03 |  | ScF5 | SSC5 | 0.02 |
| PF6 | PF8 | 0.04 |  | SoF1 | ScF3 | 0.03 |  | PF2 | SSC6 | 0.02 |
| EF2 | EF4 | 0.04 |  | SoF2 | ScF4 | 0.03 |  | PF4 | SSC6 | 0.02 |
| PF5 | SoF1 | 0.04 |  | SoF3 | SSC1 | 0.03 |  | SoF2 | SSC6 | 0.02 |
| EF2 | SoF1 | 0.04 |  | EF3 | SSC2 | 0.03 |  | EF5 | SSC8 | 0.02 |
| PF5 | SoF2 | 0.04 |  | PF2 | SSC4 | 0.03 |  | SSC2 | SSC8 | 0.02 |
| EF1 | SoF3 | 0.04 |  | PF7 | SSC5 | 0.03 |  | PF4 | PF8 | 0.01 |
| PF4 | SoF4 | 0.04 |  | EF1 | SSC5 | 0.03 |  | PF3 | EF4 | 0.01 |
| SoF3 | SoF5 | 0.04 |  | ScF3 | SSC5 | 0.03 |  | PF3 | EF5 | 0.01 |
| PF5 | ScF1 | 0.04 |  | ScF5 | SSC8 | 0.03 |  | PF6 | EF5 | 0.01 |
| EF3 | ScF1 | 0.04 |  | EF1 | EF4 | 0.02 |  | PF1 | SoF2 | 0.01 |

**S3 Data. Edge weights (continued)**

| From | To | Weight |  | From | To | Weight |  | From | To | Weight |
| --- | --- | --- | --- | --- | --- | --- | --- | --- | --- | --- |
| EF5 | SoF2 | 0.01 |  | SoF3 | SSC8 | 0.01 |  | PF1 | SSC4 | -0.02 |
| PF5 | SoF3 | 0.01 |  | SoF4 | SSC8 | 0.01 |  | SoF4 | SSC4 | -0.02 |
| PF7 | SoF3 | 0.01 |  | ScF4 | SSC8 | 0.01 |  | PF5 | SSC6 | -0.02 |
| PF6 | SoF4 | 0.01 |  | PF3 | EF1 | -0.01 |  | SoF5 | SSC6 | -0.02 |
| EF5 | SoF4 | 0.01 |  | PF2 | EF4 | -0.01 |  | SSC4 | SSC6 | -0.02 |
| PF5 | SoF5 | 0.01 |  | PF4 | SoF1 | -0.01 |  | PF2 | SSC7 | -0.02 |
| EF2 | SoF5 | 0.01 |  | PF8 | SoF4 | -0.01 |  | EF4 | SSC7 | -0.02 |
| SoF1 | ScF1 | 0.01 |  | EF2 | ScF2 | -0.01 |  | SoF5 | SSC7 | -0.02 |
| PF2 | ScF2 | 0.01 |  | SoF3 | ScF2 | -0.01 |  | ScF1 | SSC7 | -0.02 |
| EF1 | ScF2 | 0.01 |  | PF5 | ScF3 | -0.01 |  | EF1 | SSC8 | -0.02 |
| EF4 | ScF2 | 0.01 |  | PF3 | SSC2 | -0.01 |  | SoF1 | SoF3 | -0.03 |
| PF3 | ScF4 | 0.01 |  | EF2 | SSC3 | -0.01 |  | PF8 | ScF1 | -0.03 |
| SoF3 | ScF4 | 0.01 |  | EF3 | SSC3 | -0.01 |  | PF2 | ScF3 | -0.03 |
| ScF1 | ScF4 | 0.01 |  | ScF3 | SSC3 | -0.01 |  | PF6 | ScF4 | -0.03 |
| PF4 | ScF5 | 0.01 |  | EF4 | SSC4 | -0.01 |  | PF3 | ScF5 | -0.03 |
| PF5 | ScF5 | 0.01 |  | EF3 | SSC5 | -0.01 |  | ScF3 | ScF5 | -0.03 |
| SoF2 | ScF5 | 0.01 |  | SoF5 | SSC5 | -0.01 |  | ScF1 | SSC1 | -0.03 |
| ScF1 | ScF5 | 0.01 |  | ScF4 | SSC5 | -0.01 |  | PF7 | SSC3 | -0.03 |
| ScF3 | SSC1 | 0.01 |  | EF2 | SSC6 | -0.01 |  | PF4 | SSC4 | -0.03 |
| ScF5 | SSC3 | 0.01 |  | PF8 | SSC7 | -0.01 |  | PF6 | SSC4 | -0.03 |
| SSC1 | SSC4 | 0.01 |  | ScF2 | SSC7 | -0.01 |  | PF3 | SSC5 | -0.03 |
| PF4 | SSC5 | 0.01 |  | ScF3 | SSC8 | -0.01 |  | SoF2 | SSC5 | -0.03 |
| EF4 | SSC5 | 0.01 |  | PF7 | SoF1 | -0.02 |  | PF8 | SSC6 | -0.03 |
| SoF4 | SSC5 | 0.01 |  | EF5 | ScF5 | -0.02 |  | ScF4 | SSC6 | -0.03 |
| ScF1 | SSC5 | 0.01 |  | PF1 | SSC1 | -0.02 |  | PF5 | SSC8 | -0.03 |
| SSC4 | SSC5 | 0.01 |  | PF1 | SSC2 | -0.02 |  | PF6 | SSC8 | -0.03 |
| PF7 | SSC6 | 0.01 |  | ScF3 | SSC2 | -0.02 |  | PF4 | EF2 | -0.04 |
| PF2 | SSC8 | 0.01 |  | SoF5 | SSC3 | -0.02 |  | PF6 | EF2 | -0.04 |

**S3 Data. Edge weights (continued)**

| From | To | Weight |
| --- | --- | --- |
| SoF3 | ScF3 | -0.04 |
| SoF1 | ScF5 | -0.04 |
| ScF4 | SSC2 | -0.04 |
| PF1 | SSC7 | -0.04 |
| SoF1 | SSC7 | -0.04 |
| PF3 | EF3 | -0.05 |
| PF6 | SSC1 | -0.05 |
| EF5 | SSC3 | -0.05 |
| SoF2 | SSC7 | -0.05 |
| SoF3 | SSC7 | -0.05 |
| PF3 | PF5 | -0.06 |
| PF1 | EF5 | -0.06 |
| SoF1 | SSC2 | -0.07 |
| SoF3 | SSC3 | -0.07 |
| ScF3 | SSC4 | -0.07 |
| PF8 | SSC5 | -0.07 |
| SoF1 | SSC5 | -0.07 |
| PF3 | SoF3 | -0.08 |
| PF5 | ScF2 | -0.08 |
| PF8 | SSC2 | -0.08 |
| SoF3 | SSC5 | -0.08 |
| EF3 | SSC1 | -0.09 |
| ScF1 | SSC8 | -0.1 |
| EF3 | ScF3 | -0.11 |
| SoF1 | SSC6 | -0.11 |
