# Supplementary material for: Pediatric health-related quality of life and school social capital through network perspectives
Source: PLoS One. 2020 Dec 2;15(12):e0242670. doi: 10.1371/journal.pone.0242670 (PMC7710098; doi:10.1371/journal.pone.0242670)
Supplement: S7 Data — (DOCX) [file pone.0242670.s007.docx]

**S7 Data. Edge weights with statistical significance by the network comparison test (4^th^ – 6^th^ graders vs 7^th^ – 9^th^ graders)**

| node 1 | node 2 |
| --- | --- |
| PF4 | SoF1 |
| EF3 | SoF1 |
| PF7 | SoF3 |
| SoF1 | SoF3 |
| SoF2 | SoF3 |
| SoF4 | SoF5 |
| PF6 | ScF2 |
| PF1 | ScF3 |
| PF6 | ScF3 |
| EF1 | ScF3 |
| EF5 | ScF4 |
| PF2 | SSC2 |
| SoF3 | SSC2 |
| SoF4 | SSC6 |
| PF1 | SSC10 |
| SSC4 | SSC10 |
